# Continuous ambulatory peritoneal dialysis (CAPD) in children: a successful case for a bright future in a developing country

**DOI:** 10.11604/pamj.2019.33.71.17042

**Published:** 2019-05-30

**Authors:** Younoussa Keita, Aliou Abdoulaye Ndongo, Cathérine Bebey Engome, Ndeye Fatou Sow, Ndiogou Seck, Lamine Thiam, Papa Malick Diouf, Ahmed Tall Lemrabott, Idrissa Basse, Abdou Niang, Saoussen Krid, Claude Moreira, Remi Salomon, Boucar Diouf, Assane Sylla, Ousmane Ndiaye

**Affiliations:** 1 Pediatric Unit, Aristide Le Dantec Hospital, Dakar, Sénégal; 2 Pediatric Unit, Regional Hospital Centre, Saint Louis, Sénégal; 3 Pediatric Unit, Regional Hospital Centre, Ziguinchor, Sénégal; 4 Nephrology Unit, Aristide Le Dantec Hospital, Dakar, Sénégal; 5 Pediatric Unit, Diamniadio Hospital, Dakar, Sénégal; 6 Nephrology Unit, Dalaldiam Hospital, Dakar, Sénégal; 7 Pediatric unit, Necker Hospital, Paris, France; 8 Pediatric Unit, Albert Royer’s Children Centre, Dakar, Sénégal

**Keywords:** Peritoneal dialysis, developing countries, child, Senegal

## Abstract

The authors report the first case of successful peritoneal dialysis (PD) in a developing country performed about a 13-year-old adolescent followed-up for stage V chronic kidney disease (CKD) with anuria. After 3 months of hemodialysis, the parents opted for continuous ambulatory peritoneal dialysis (CAPD) as they wished to return home located 121km from Dakar. After PD catheter insertion, the plan proposed to the patient consisted 3-4 hours stasis of isotonic dialysate during the day and a night stasis of 8 hours of icodextrin for an injection volume of 1L per session. The patient and his mother were trained and assessed on the PD technique. After dialysis adequacy was tested while hospitalised, they were able to return home and continued the sessions following the same plan prescribed and while keeping in touch, by telephone, with the medical team. The technique assessment at the day hospital every 2 weeks revealed dialysis adequacy and satisfactory tolerance of PD at home after 04 months of observation. It was the first case of successful CAPD in the pediatrics unit in this context. Scaling this technique is a challenge for the pediatric nephrologist in developing countries like Senegal.

## Introduction

Management of chronic kidney disease remains a challenge in developing countries due to the absence of kidney transplant, limited access to extra-renal purification and the low involvement of health policies particularly for children [1, 2]. PD remains a reliable alternative for renal replacement in pediatrics both in acute and chronic situations. The two PD modalities are represented by continuous ambulatory peritoneal dialysis (CAPD) and automated peritoneal dialysis (APD) with their variants [3-5]. The authors report the first case of successful CAPD in pediatrics in a 13-year-old Senegalese, resident 121km from Dakar.

## Patient and observation

**Figure 1:**
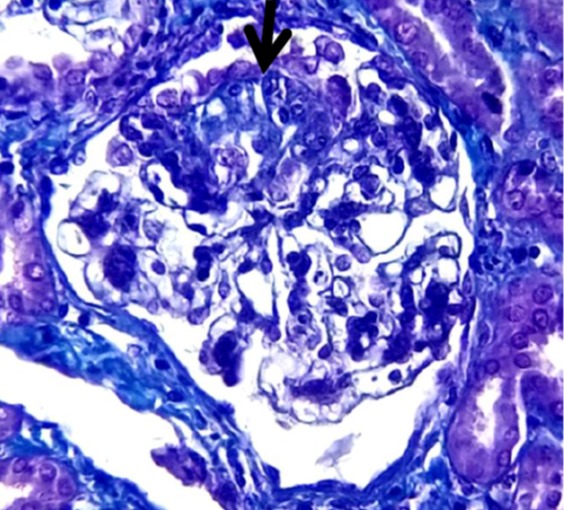
Focal and segmental glomerulosclerosis (FSGS): fibrohyaline segmental glomerular lesions surrounded by dysmorphic podocytes Masson’s trichrome x 250

**Figure 2:**
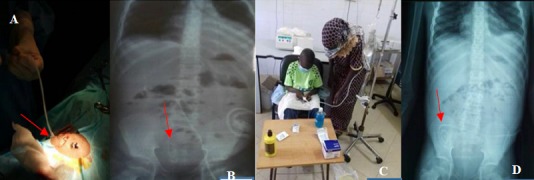
A) surgical insertion of TENCKOFF catheter; B) control ASP showing the catheter in a good position in the pouch of Douglas; C) the patient and his mother after the drainage; D) catheter migration in the right lumbar region

This was a 13-year-old boy monitored in the unit since 2017-10-04 for stage V Chronic Kidney Disease (CKD) following a primary nephrotic syndrome by Segmental and Focal Glomerulosclerosis (FSGS) (Figure 1) cortico-resistant and cyclo-resistant since he was 11 years old. The patient was anuric with diuresis at 0.19ml/kg/h. He weighed 32kg and measured 131cm with a normal BMI for his age. He had delayed pubertal development (P1G3). Faced with signs of intolerance in uremia, he first received two successive sessions of haemodialysis then three sessions per week for over 3 months in an adult center. He was switched to CAPD following his parents' choice motivated by a desire to return home situated 121km from Dakar. The TENCKOFF (Figure 2 A, B) catheter was inserted on 2018-03-05 in urology in collaboration with the pediatrics and adult nephrology team. On day 1 after catheter insertion, the peritoneal cavity was rinsed. On day 2 after insertion, the patient had a fever at 39oC, abdominal pain and greenish vomiting. Upon examination, he would scream when his navel is touched. Analysis of the drainage liquid had revealed 650 leucocytes/mm^3^ with 62% of neutrophils. The dialysate culture was sterile. The hemoculture came back negative. The nasal swab isolated *Staphylococcus aureus*. The patient received 1g of C3G in each bag of 2L of dialysate associated with a bacitracin+neomycine nasal rub as from day 2 after catheter insertion. The plan proposed to the patient was 4 swabs a day (8am - 12noon - 4pm - 8pm - 8am) with a stasis of isotonic dialysate of 4 hours per session during the day and a stasis of icodextrin for 8 hours at night. The total time for dialysis was 24 h/day and the volume of injection per cycle was 1L of dialysate. The peritoneal control fluid at day 7 was sterile (leucocytes 21 elements/mm^3^ with 95% lymphocytes). The antibiotic treatment was done by oral route for 14 days. Besides the PD, the patient was receiving anti-hypertensive treatment, water restriction, active calcium and vitamin D supplementation, erythropoietin and a CKD diet. After the infection period, an equilibration Test (PET test) revealed a Hyper-permeable peritoneal membrane with D/P creatinine at 0.91. The patient and his mother were trained and assessed on the PD technique (Figure 2 C). By the end of a month, they had become independent and could recognize the signs or dysfunction of the catheter. Patient assessment during this first month of dialysis revealed an adequate clinical and biological condition (Table 1). The patient and his mother returned home at Bambey, a town situated 121km from Dakar with a tracking sheet in which the weight, blood pressure, temperature and ultra filtrate are noted. After a second month of observation, the patient complained of abdominal pain at the day hospital. The plain abdominal X-ray revealed a catheter migration to the right (Figure 2 D). The catheter was repositioned in the patient through the use of laxatives. After 4 months of observation, the dialysis was considered adequate on a clinical and biological basis (Table 1).

**Table 1:** Evolution of monitoring parameters for dialysis adequacy

Monitoring parameters	Before CAPD	M1	M2	M3	M4
Blood urea (g/L)	1.45	0.97	0.79	1	0.69
Creatininaemia (mg/L)	94.1	82.8	81.1	79.9	114.8
Haemoglobin (g/dL)	6.6	5.6	7.7	11	8.9
Natremia (mEq/L)	134	136	132	133	130
Kalemia (mEq/L)	6.7	5	5	5.1	4.8
Calcaemia (mg/L)	114.36	90.14	93	95	79.77
Phosphoremia (mg/L)	87.2	47.9	54.5	38.5	38.1

M=Month; CAPD = continuous ambulatory peritoneal dialysis

## Discussion

Peritoneal dialysis remains the chronic kidney disease dialysis replacement therapy at this dialysis stage in children in this context. It is a back-up solution in children with ESRD though with significant constraints [6, 7]. Even though kidney transplant is the preferred treatment method for patients suffering from CKD, most patients are placed on dialysis while waiting for transplant or as the only treatment [3]. The patient went in for CAPD, which, in this context, was the only method left for the child to be able to return home with the family. Clinically, the plan prescribed helped with significant regression of the edematous-ascitic syndrome with weight loss, positive ultra filtration and blood pressure control with a Cardiothoracic Index at 0.53. According to the authors, the short stasis during the day facilitated ultra filtration while the night stasis of icodextrin facilitated purification [8]. The challenge of dialysis adequacy in this patient also laid on the fact that residual diuresis was practically nil. In fact, only residual renal function and peritoneal ultra filtration (UF) maintainers in anuric patients were correlated to the survival of patients and considered as major predictive factors of cardiovascular morbidity and mortality [9, 10]. These two factors then became essential parameters for adequacy. The management of anaemia was one of the difficulties faced with the patient due to the expensive cost of erythropoietin even if consumption is less in PD than in hemodialysis [11]. Moreover, calcium phosphate metabolism control plays a significant role in PD adequacy and especially glycemic load control considered as the cornerstone in PD patients [9]. The patient had normoglycemia and a satisfactory nutritional status. The authors explain that among other factors, a good nutritional status was also very essential for PD adequacy [12-14]. Nutritional, social and psycho-emotional support was proposed to the child and his family especially through the social worker of the unit.

Among the PD complications, dialysis fluid infection is the most common, first reason why the technique can be stopped with an average period of 20 to 30 months-patient [15]. In this context of a developing country, prevention of peritoneal liquid infections is our bedrock. Strict asepsis, prophylactic antibiotic therapy during catheter insertion and education of care givers and the patient will minimise the risk of peritonitis. Despite a hyper-permeable dialysis membrane after the infectious period, the dialysis plan did not change. In fact, the dialysis at this moment was adequate. This hyper-permeability could be explained by the recent dialysis fluid infection. According to the authors, the peritoneal equilibration test (PET test) should be done during observation of the patient, in case of inadequacy and far from the peritonitis periods. After the infection, the child had a catheter migration two months after the onset of PD. The authors mentioned in the literature that complications related to PD catheter displacement occur late and are associated with abdominal pain [4]. In this patient, the catheter was repositioned by taking a laxative. After 4 months of observation, the patient and his mother were still independent with CAPD which helped solved the problem of lodging this family in Dakar. Ever since, the patient was staying at home with his family 121km from Dakar. He narrated his story during several cultural events both at the family and social level. He will be able to return to school next academic year in the month of October. These elements sum up the many advantages of this technique in this patient amongst others as mentioned in the literature [16]. For a better future, kidney transplant authorised in Senegal since 2015 would be the ideal treatment for this boy.

## Conclusion

The authors report on their first successful CAPD experience in children in Senegal. This technique helped to stabilise the patient. Satisfaction in this case laid on the one hand in overcoming the constraints linked to chronic hemodialysis and on the other hand in the return of this child to his family. Although this method of extra renal purification was effective, it is important to underline that much remains to be done to prevent the risk of infection and to popularise PD in this context of a developing country. For the future of this patient, a kidney transplant would be the best therapy.
